# Efficacy of Theobromine and Its Metabolites in Reducing the Risk of Uric Acid Lithiasis

**DOI:** 10.3390/ijms241310879

**Published:** 2023-06-29

**Authors:** Antonia Costa-Bauzá, Paula Calvó, Yumaira Hernández, Fèlix Grases

**Affiliations:** 1 Laboratory of Renal Lithiasis Research, University Institute of Health Sciences Research (IUNICS-IdISBa), University of Balearic Islands, 07122 Palma de Mallorca, Spain; paula.calvo@uib.es (P.C.); fgrases@uib.es (F.G.); 2 Urology Service of Manacor Hospital, 07500 Manacor, Spain; yhernandez@hmanacor.com

**Keywords:** uric acid lithiasis, theobromine, methylxanthines, crystallization inhibitors, lithiasis risk

## Abstract

Uric acid lithiasis accounts for about 10% of all types of renal lithiasis. The most common causes of uric acid lithiasis are low urinary pH, followed by high concentration of urinary uric acid, and low diuresis. Treatment of patients consists of alkalinization of urine, reducing the consumption of purine-rich foods, and administration of xanthine oxidase inhibitors, because there are no established therapeutic inhibitors of uric acid crystallization. We recently found that theobromine inhibited uric acid crystallization in vitro, and that the increased urinary level of theobromine following its oral consumption was associated with the prevention of uric acid crystallization. In this study, we evaluated the inhibitory effects of theobromine metabolites and other methylxanthine-related compounds on uric acid crystallization. We also measured the urinary concentrations of theobromine and its metabolites in samples from healthy individuals and patients with uric acid stones and compared the extent of uric acid supersaturation and uric acid crystal formation in these different samples. Theobromine and other methylxanthines that lacked a substituent at position 1 inhibited uric acid crystallization, but other methylxanthines did not have this effect. Individuals with clinical parameters that favored uric acid crystallization did not develop uric acid crystals when theobromine and its metabolites were in the urine at high levels. Thus, theobromine and its metabolites reduced the risk of uric acid lithiasis.

## 1. Introduction

Renal lithiasis has a worldwide prevalence of about 10% [1,2,3], and the prevalence has increased over time [3,4]. A 2008 study estimated that the prevalence of renal lithiasis in 2050 could exceed 30% in some geographic areas due to increases in ambient temperature [5]. Kidney stones can have different compositions, and about 10% of them consist of uric acid (UA) [6,7,8,9]. A high percentage of patients with UA lithiasis also have comorbidities such as hyperglycemia, obesity, and metabolic syndrome [8,9,10]. 

There are different types of renal stones, and these different stones have different etiologies. Stone formation can be affected by metabolic factors, genetic factors, and lifestyle factors, including diet and drug use. Urinary alterations and physiochemical factors also affect the risk of crystallization. An important etiological factor is urinary supersaturation of compounds that readily crystallize.

The main etiologic factors associated with the development of UA stones are urinary pH below 5.5, hyperuricosuria (defined as 24 h urinary UA exceeding 750 mg/day in females and 800 mg/day in males), and low diuresis [9,11,12]. These conditions have different possible causes but are generally related to lifestyle [13]. For example, low fluid intake is the most common cause of low diuresis, and this leads to increased urinary UA concentration (values greater than 500 mg/L) and eventually supersaturation. The consumption of foods with abundant purines, such as red meat, fish, shellfish, and to a lesser extent legumes, can cause hyperuricosuria because the body’s catabolism of purine produces UA as the final product. Certain drugs that inhibit renal tubular reabsorption of UA or increase purine catabolism can also cause hyperuricosuria. Finally, a urinary pH below 5.5, which may be due to chronic diarrhea or high production of endogenous acids due to the consumption of a high-protein diet, also increases the risk for UA stones. However, many patients with UA lithiasis do not present with any of these characteristics, and recent studies found that patients with metabolic syndrome often present with a low urinary pH, regardless of diet [10]. The use of certain drugs, such as thiazides or furosemide, also decreases the urinary pH [14].

Although the major etiological factors associated with UA lithiasis are low urinary pH, followed by high urinary levels of UA, and low diuresis, many individuals who have elevated urinary UA levels or low urinary pH do not develop UA stones [15]. This indicates that other factors also affect the formation of these stones. In fact, the presence of certain kinetic factors can affect crystallization, and these include heterogeneous nucleants that facilitate crystallization and crystallization inhibitors [16,17].

The traditional treatments for UA lithiasis are alkalinization of urine, restricting the consumption of purine-rich foods, and pharmacological treatment with a xanthine oxidase inhibitor [18]. In contrast to calcium lithiasis, there are no available therapeutic agents that inhibit the crystallization of UA. We recently found that theobromine, a dimethylxanthine alkaloid that is abundant in cocoa, inhibits UA crystallization [19] and therefore may be useful for the prevention of UA lithiasis. In order to verify this usefulness, we carried out a comparative study of the treatment of UA lithiasis with citrate versus citrate+theobromine [20]. In particular, our results confirmed that dietary theobromine increased urinary theobromine excretion, and that this was related to a decreased risk of UA crystallization. Nevertheless, the theobromine urinary concentrations found under citrate+theobromine treatment (0.102 ± 0.083 mmol/L) [20] were in the low limit of efficacy (0.055 mmol/L) [19], and for some patients, theobromine urinary concentrations were much lower than 0.055 mmol/L, which does not allow justification of the observed protective effect of theobromine against AU crystallization.

After intake of theobromine, the human body metabolizes it and excretes different products into the urine, mainly 7-methylxanthine (36%), unmetabolized theobromine (21%), 3-methylxanthine (21%), and 3,7-dimethyluric acid (1.3%) [21]. 

Thus, in order to better characterize the effects of methylxanthine consumption in the prevention of UA lithiasis, one objective of this study was to evaluate the inhibitory effects of theobromine metabolites and other methylxanthine-related compounds such as pentoxifylline (used as a drug to treat muscle pain in people with peripheral artery disease), dyphylline (used in the treatment of respiratory disorders such as asthma), and 7-(β-hydroxyethyl)theophylline on UA crystallization in vitro. A second objective was to measure the urinary concentrations of theobromine and its metabolites in healthy individuals and in patients with UA stones, and to relate them to UA supersaturation and UA crystal formation in urine samples from these different individuals.

## 2. Results

### 2.1. Turbidimetric Assay with Synthetic Urine Samples

**Figure 1:**
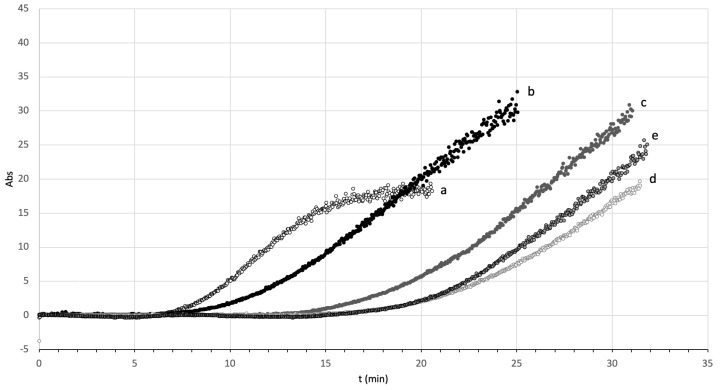
Effect of the mixing time of synthetic urine with theobromine prior to the induction of UA supersaturation on the kinetics of UA crystallization followed by turbidimetry. A control sample was incubated without theobromine (a); other samples were incubated with 0.2 mM theobromine and were mixed for 0 min (b), 5 min (c), 15 min (d), or 30 min (e).

We first measured the effect of the mixing time of synthetic urine with theobromine prior to the induction of UA supersaturation (Figure 1). The results demonstrated that the time needed for induction of UA crystals increased as the mixing time increased, but there was very little difference for mixing times of 15 min and 30 min.

**Figure 2:**
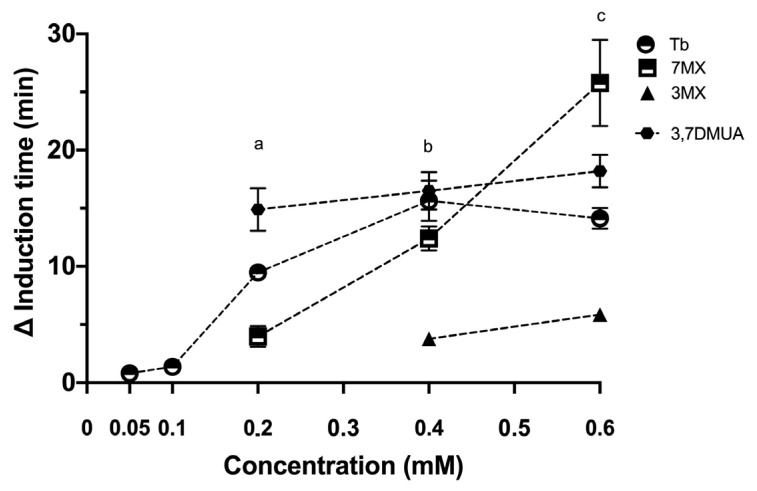
Effect of the concentration of theobromine and related methylxanthines on the induction time of UA crystallization in synthetic urine at pH 4.65. Induction times were obtained from crystallization curves (Figure 1), and all results are expressed as the increment of the induction time with respect to the induction time of the control (Δ induction time) and are means of triplicates ± standard deviations. (a) Statistical differences between the different compounds at 0.2 mM concentration (*p* < 0.05). (b) Statistical differences between 3MX and the other compounds at 0.4 mM concentration (*p* < 0.05). (c) Statistical differences between 7MX and the other compounds at 0.6 M concentration. Tb: theobromine; 7MX: 7-methylxantine; 3MX: 3-methylxantine; 3,7DMUA: 3,7-dimethyluric acid.

The addition of 1-methylxanthine, pentoxifylline, 7-(β-hydroxyethyl)theophylline, and dyphylline at concentrations of 0.60 mM had no significant effect on the time for induction of crystallization. However, under conditions of UA supersaturation (SS_UA_ = 3.55), 0.60 mM 7-methylxanthine, 3,7-dimethyluric acid, and 3-methylxanthine inhibited crystallization; the first two of these compounds were about as effective as theobromine, but 3-methylxanthine had a weaker effect (Figure 2). 

**Figure 3:**
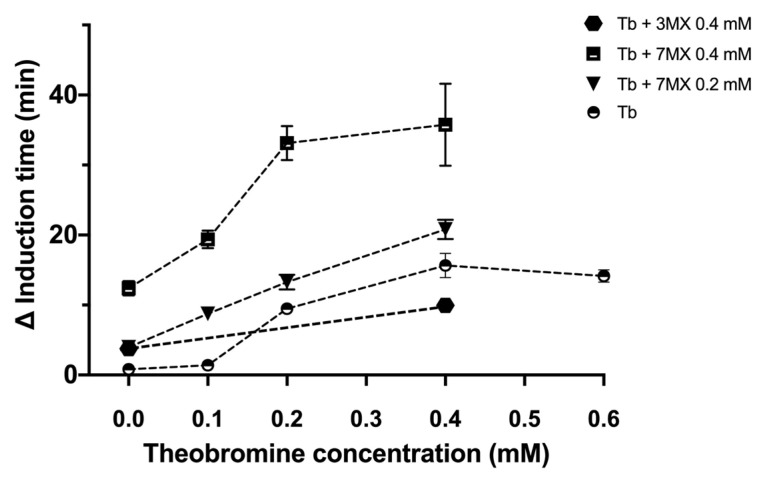
Effect of the combination of theobromine with different methylxanthines on the induction time of UA crystallization in synthetic urine at pH 4.65. All results are expressed as the increment of the induction time with respect to the induction time of the control (Δ induction time) and are means ± standard deviations of triplicates.

We then examined the effects of the combined presence of theobromine with two methylxanthines (3-methylxanthine and 7-methylxanthine) on the time for induction of UA crystallization (Figure 3). We did not test 3,7-dimethyluric acid because its level in urine is only 6% that of theobromine. The results showed that the combination of theobromine with 7-methylxanthine had a stronger effect, and this effect appeared to increase as the concentration of 7-methylxantine increased.

**Figure 4:**
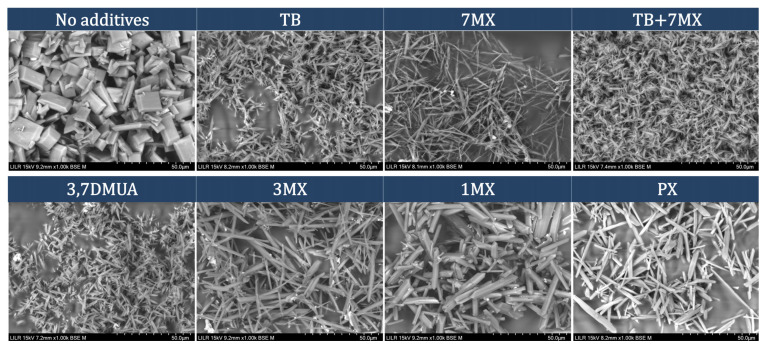
Scanning electron microscopy of UA crystals formed in artificial urine collected after the turbidimetric assay. Top row: no additives; 0.60 mM of theobromine (Tb); 0.60 mM 7-methylxanthine (7MX); mixture of 0.4 mM Tb with 0.40 mM 7MX; bottom row: 0.60 mM 3,7-dimethyluric acid (3,7DMUA); 0.60 mM 3-methylxanthine (3MX); 0.60 mM 1-methylxanthine (1MX); or 0.60 mM paraxanthine (PX).

We then examined the effect of the different tested methylxanthines on morphology of UA crystals formed in artificial urine at pH 4.65. Scanning electron microscopy showed that the presence of all the methylxanthines altered the morphology of crystals, in that all obtained samples had thinner and longer crystals (Figure 4) in contrast with the crystals obtained in their absence, which had rectangular prism form. 

### 2.2. Urine Samples from Healthy Individuals and Patients with UA Stones

**Figure 5:**
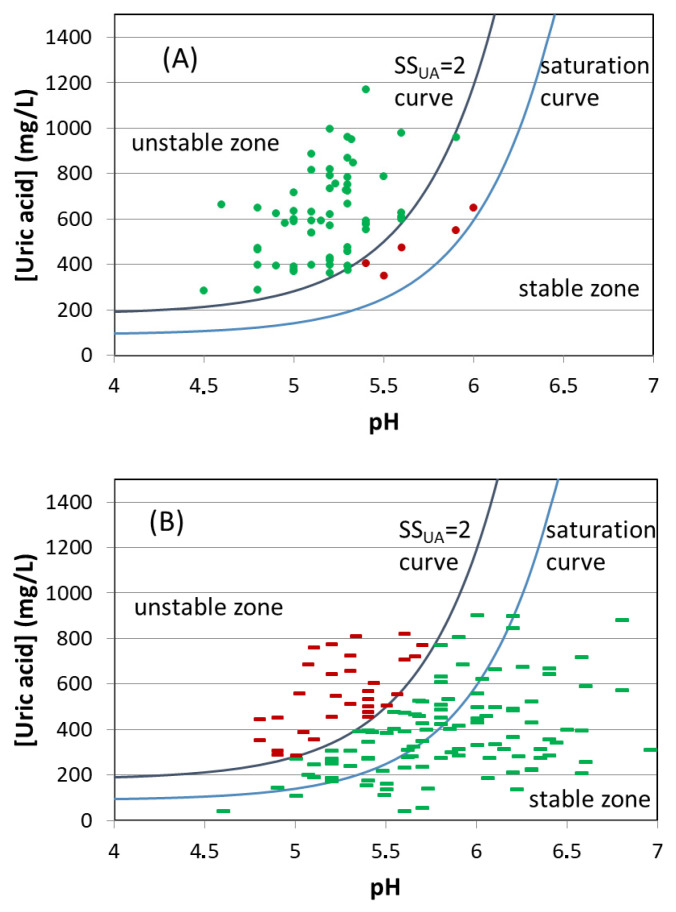
Urinary pH and UA concentration of individual urine samples, and UA saturation curves (SS_UA_ = 1) and supersaturation curves (SS_UA_ = 2). (**A**) Samples with positive UA crystallization results, with true positives in green and false positives in red. (**B**) Samples with negative UA crystallization results, with true negatives in green and false negatives in red.

We then applied the UA crystallization test (RUAC) in 60 urine samples from 20 healthy volunteers and 153 urine samples from 54 UA stone formers. There were 66 samples that had a risk for crystallization (Figure 5A) and 147 samples that did not have a risk for crystallization (Figure 5B). The results showed that only five samples with a risk for crystallization were false positive results (red in Figure 5A), but thirty-four samples with no risk for crystallization were false negatives (red in Figure 5B). These classifications were based on curves for saturation (SS_UA_ = 1) and supersaturation (SS_UA_ = 2) that were calculated as previously described [8,22].

**Figure 6:**
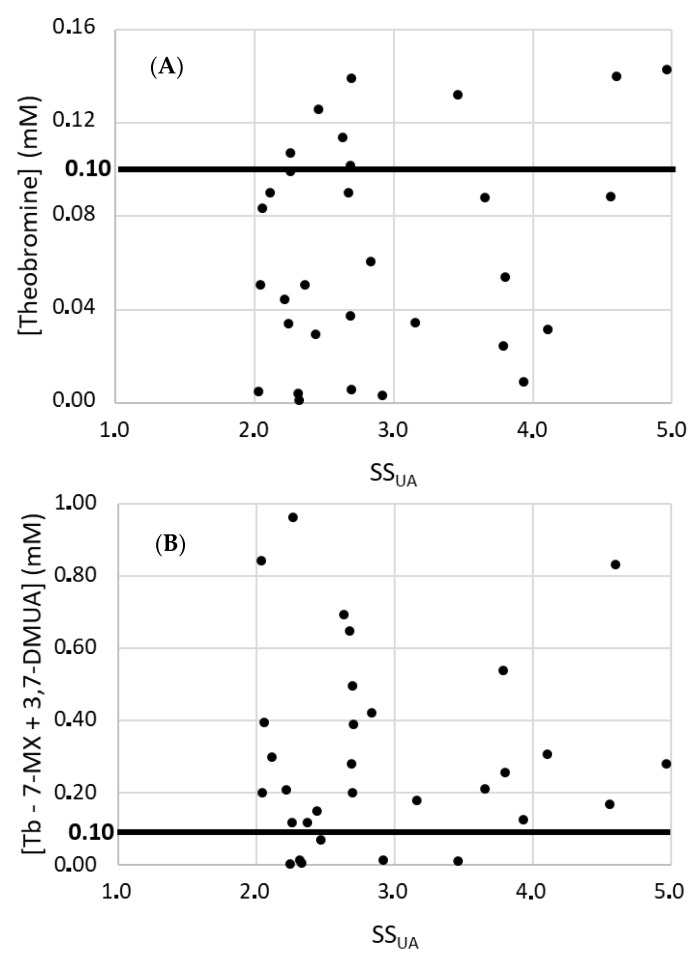
Urinary theobromine concentration (**A**) and sum of theobromine, 7-methylxanthine, and 3,7-dimethyluric acid concentrations (**B**) in the 34 urine samples with SS_UA_ greater than 2 for which the UA crystallization test was negative (See Figure 5B). Tb: theobromine; 7-MX: 7-methylxanthine; 3,7-DMUA: 3,7-dimethyluric acid.

We also measured the concentration of theobromine (Figure 6A) and of the sum of theobromine + 7-methylxanthine + 3,7-dimethyluric acid (Figure 6B; compounds with similar inhibitory effects on UA crystallization) of the 34 samples with false negative results.

**Figure 7:**
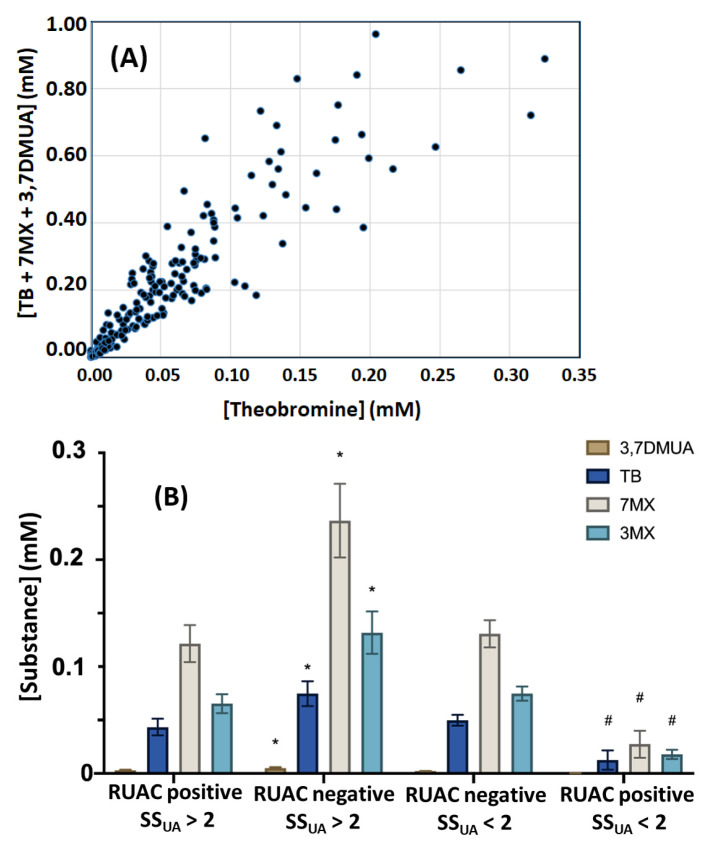
Relationship of the concentration of theobromine with the concentration of theobromine and metabolites that had similar inhibitory effects on UA crystallization (**A**) and concentration of each compound (mean ± standard error of mean) in urine samples with different results for RUAC test and SS_UA_ > 2 or SS_UA_ < 2 (**B**). * Significant differences with the “RUAC positive with SS_UA_ > 2” group, *p* < 0.005; # Significant differences with the “RUAC negative with SS_AU_ < 2” group, *p* < 0.05. TB: theobromine; 7MX: 7-methylxanthine; 3MX: 3-methylxanthine; 3,7DMUA: 3,7-dimethyluric acid.

Further analysis showed that the concentration of theobromine and its metabolites (7-methylxanthine and 3,7-dimethyluric acid) was nearly two-and-a-half times higher than the concentration of theobromine alone (Figure 7A) and that those samples with SS_UA_ greater than two for which the UA crystallization test was negative had the highest urinary concentrations of theobromine and its metabolites, while those samples with SS_UA_ lower than two for which the UA crystallization test was positive had the lowest ones (Figure 7B).

## 3. Discussion

The obvious effect of mixing time prior to the induction of UA supersaturation on the inhibitory effects of theobromine (Figure 1) suggests that this inhibition is related to an interaction between theobromine and UA at the crystalline interface and in solution. In fact, a previous study of the molecular interactions between theobromine and UA in solution found that these molecules had a π-stacking interaction [23]. The formation of theobromine–UA clusters would decrease the supersaturation of UA and thus delay the formation of UA crystals. Our results demonstrated that the formation of UA crystals was delayed when the mixing time was 15 min or longer (Figure 1).

Our comparison of the effects of different methylxanthines showed theobromine, 7-methylxanthine, 3,7-dimethyluric acid, and 3-methylxanthine inhibited UA crystallization. However, none of the tested xanthine derivatives with substituents at position 1, 1-methylxanthine, pentoxifylline, 7-(β-hydroxyethyl)theophylline, and dyphylline, were inhibitors of UA crystallization under the conditions studied. This finding agrees with previous results, which reported that caffeine, paraxanthine, and theophylline, each of which has a methyl group at position 1, do not inhibit UA crystallization [19]. Moreover, we found that compounds with the greatest inhibition of UA crystallization had a methyl group at position 7. Thus, for xanthines, the positions of different substituent groups appear to affect the capacity to inhibit UA crystallization.

Two prospective clinical analyses of dietary risk factors for kidney stones found that caffeine consumption was inversely associated with this risk [24,25], even though caffeine itself does not inhibit UA crystallization. This may be because caffeine intake by humans leads to the urinary excretion of dimethylxanthines, methylxanthines, and dimethyl and methyluric acids [26], and the total concentration of these metabolites is expected to be sufficient for inhibition of UA crystallization, based on the results presented herein.

Consumption of theobromine, which is abundant in cocoa, is excreted in the urine mainly as 7-methylxanthine (36%), unmetabolized theobromine (21%), 3-methylxanthine (21%), and 3,7-dimethyluric acid (1.3%) [21], although these same compounds also occur in urine following caffeine consumption. For this reason, we tested the effects of mixtures of theobromine with 7-methylxanthine and 3-methylxanthine (Figure 3). The results showed that the total inhibitory effect was greater than or equal to the sum of the effects of each individual compound, with some evidence of synergistic effects at higher concentrations. Thus, UA crystallization in urine is affected by the global concentration of theobromine and several of its metabolites, especially 7-methylxanthine and 3,7-dimethyluric acid, which may also come from the metabolism of caffeine. 

UA solubility obviously depends on pH (Figure 5). Thus, solubility is below 110 mg/L for a urinary pH below 5.0, below 250 mg/L at pH 5.5, and higher than 600 mg/L for a pH over 6.0. Consequently, UA supersaturation in urine is a function of urinary pH as well as UA concentration [8].

Crystal formation is only possible in a supersaturated solution (SS > 1), although there is a range of supersaturation values at which a medium is metastable, in which crystals already formed can grow, but new crystals do not form. For supersaturation values above the upper limit of the metastable zone, the medium becomes unstable, and crystallization is spontaneous. For UA in urine, this limit corresponds to a SS_UA_ level above 2 [8,22], even though certain factors can modify the kinetics of the process increasing the metastable range.

The risk of UA crystallization in urine samples, determined as explained above, was positive for samples with UA supersaturation values higher than 2 (Figure 5A), and there was no risk of crystallization in samples with supersaturation values below 2 (Figure 5B). However, we found that 34 of the 147 samples with no risk for UA crystallization had supersaturation values greater than 2; these samples should have crystallized, but they did not. Heterogeneous nucleants reduce the supersaturation value required for nucleation of new crystals. The presence of such factors in urine may explain why some of our samples developed UA crystals even when the SS_UA_ was below 2 (Figure 5A). However, this situation was not frequent (five of sixty-six samples).

Conversely, inhibitors of crystallization retard or impede one or more different steps of the crystallization process, and the presence of these inhibitors increases resistance to crystallization when the SS is above the metastable limit. This phenomenon seemed to be more common in our samples (Figure 5B), indicating that many of our urine samples contained substances that prevented UA crystallization.

Analysis of the urinary theobromine concentration of samples in which UA crystallization did not occur when the supersaturation exceeded 2 showed that most of them had a theobromine concentration below 0.1 mM (Figure 6A). This is the lowest theobromine concentration at which there was inhibition of crystallization for UA supersaturation of 3.55. Nevertheless, the total concentration of theobromine and its metabolites was usually higher than 0.1 mM (Figure 6B). Thus, theobromine and its metabolites are likely responsible for preventing UA crystallization under these conditions. These results show that with a low intake of theobromine, crystallization inhibition values of the order of three times higher than expected, if only urinary theobromine concentration is considered, can be achieved. However, we have to consider that some samples with SS_UA_ above 2 that had very low concentrations of theobromine and metabolites did not form UA crystals. This suggests that other heretofore unidentified inhibitors were also present in the urine samples.

## 4. Materials and Methods

### 4.1. Reagents and Solutions

**Figure 8:**
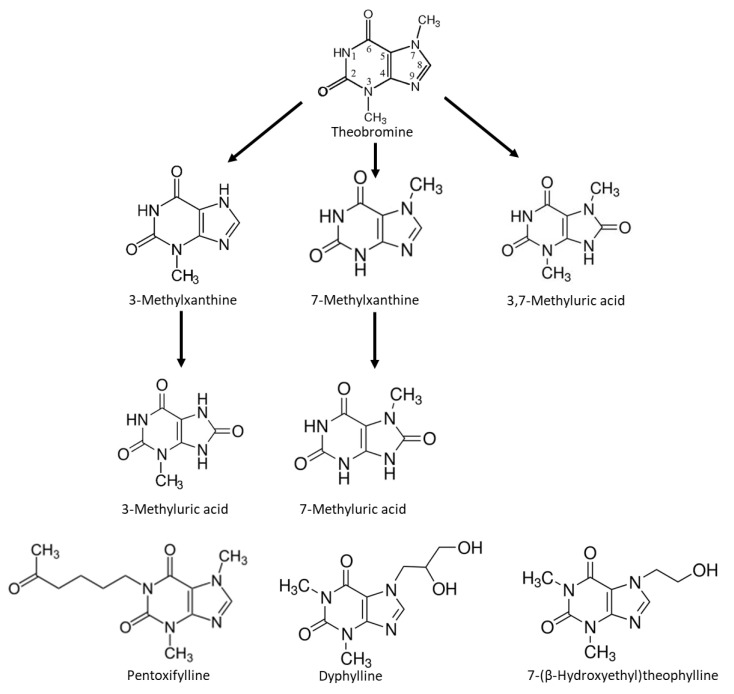
Chemical structures of theobromine, theobromine metabolites, and related methylxanthines: pentoxifylline (3,7-dimethyl-1-(5-oxohexyl)xanthine), dyphylline (7-(2,3-dihydroxypropyl)theophylline), and 7-(β-hydroxyethyl)theophylline (1,3-dimethyl-7-(2-hydroxyethyl)xanthine). Note the presence or absence of different substituents at position 1 and the presence or absence of a methyl group at position 7 in the different molecules.

UA, theobromine, 1-methylxanthine, 3-methylxanthine, 7-methylxanthine, 3,7-dimethyluric acid, pentoxifylline, 7-(β-hydroxyethyl)theophylline, and dyphylline (Figure 8) were purchased from Sigma-Aldrich (St. Louis, MO, USA). Synthetic urine components were obtained from Panreac (Montcada i Reixac, Barcelona, Spain). Chemicals of analytical reagent-grade purity were dissolved in ultra-pure deionized water from a Milli-Q system and passed through a 0.45 µm filter before use. A UA stock solution (2 g/L) was prepared daily by dissolving 1 g of UA in 0.5 L of water, followed by addition of NaOH to achieve a final pH of 10.70. Synthetic urine (Table 1) was prepared without calcium or oxalate to prevent calcium oxalate crystallization. The pH of the solution was adjusted to 5.40 before use.

### 4.2. Turbidimetric Assay

A previously described turbidimetric assay was used to determine the effect of theobromine and its metabolites on UA crystallization in an artificial urine medium, which contained 400 mg/L of UA and had a pH of 4.65 [19]. These conditions correspond to supersaturation of UA (SS_UA_ = 3.55). The turbidimetric system consisted of a photometer (AvaSpec-ULS2048CL-EVO, Avantes, The Netherlands) that was equipped with a fiber-optic light-guide measuring cell that was attached to a light path (2 × 10 mm) reflector. Crystallization was assessed at a constant temperature (37 °C) via mixing using a magnetic stir bar (300 rpm). 

A stock solution of synthetic urine (80 mL) was added to a crystallization flask, followed by the addition of 80 mL of water and 40 mL of a 2 g/L UA solution. Then, 0.5 M HCl was added until the pH reached 4.65 to achieve UA supersaturation. This pH value was selected to ensure a short crystallization time (6–10 min without an inhibitor). Absorbance was recorded continuously to monitor crystal formation. 

The studied compounds (Figure 8) were assayed at concentrations of 0.05 to 0.60 mM. These concentrations were achieved by adding an appropriate volume of a 2 mM solution of each compound to the crystallization mixture (with a corresponding reduction in the volume of added water) prior to the addition of HCl. This solution was mixed with a magnetic stir bar (300 rpm) for 15 min before induction of UA supersaturation. 

Induction time (ti) was set as the time when the absorbance first began to increase. The effects of tested substances on the crystallization of UA were expressed as the increment of the induction time with respect to the induction time of the control (Δ induction time). Each experiment was performed in triplicate.

For compounds that inhibited crystallization, the effects of different combinations were also determined.

### 4.3. Structural Analysis of Crystals

The morphologies of the UA crystals that formed in synthetic urine in the absence or presence of the different tested compounds were examined using a scanning electron microscopy system (SEM, Hitachi S-3400N, Tokyo, Japan) coupled with XR energy dispersive microanalysis (Bruker AXS XFlash Detector 4010, Berlin, Germany). Crystals were collected at the end of each experiment by passing the solution through a 0.45 µm filter. They were then dried in a desiccator and examined using SEM by placing the crystals on a sample holder with fixation on adhesive conductive copper tape.

### 4.4. Participants

Urine samples were provided by 20 healthy adult volunteers (11 males and 9 females, mean age: 37 years, age range: 22 to 65 years) and 54 volunteers who were active formers of UA stones (43 males and 11 females, mean age: 60 years, age range: 43 to 75 years). All volunteers were from Mallorca, Spain, and individuals with urinary infections were excluded. 

The study design was approved by the local Ethics Investigation Committee of the Balearic Islands (IB 3475/17 PI) and by the Research Committee of Hospital Manacor and the Research Ethics Committee of Balearic Islands [CEI-IB] (IB3414). All participants provided written informed consent before participation. 

### 4.5. Urine Samples

Three spot urine samples were collected from each participant at intervals of at least 7 days. Samples were collected under standard conditions in a sterile container, with no additives or preservatives, as they were processed immediately after collection. All participants were instructed to follow their usual diets. Patients with UA stones received oral theobromine (120 mg/day) for 14 days before collection of the third sample, and the results are included in a previously published study [20] of dietary intervention to assess the efficacy of theobromine in UA stone prevention.

The main urinary parameters related to UA lithogenesis were determined. Thus, urinary pH was measured using a Crison pH meter, and UA concentration was determined using the uricase method. Theobromine and its metabolites were measured using ultra-high-performance liquid chromatography and high-resolution mass spectrometry (UHPLC/HRMS), as previously described [27].

The urinary pH and UA concentrations were used to determine the UA supersaturation (SS_UA_) of a sample. As previously described [20], the solubility (S) and SS_UA_ were calculated using the following formulas at the pH of the sample:

$$S=\frac{K_{\mathrm{sp}}}{K_{a1}}\;\left[1+\frac{K_{a1}}{10^{-\mathrm{pH}}\gamma_{{\mathrm{HU}}^{-}}}\right]\;\;\;{\mathrm{SS}}_{\mathrm{UA}}=\frac{c\left(H_{2}U\right)}{S}$$

where S (mol/L) and SS_UA_ are at the pH of the sample, K_sp_ is the solubility product of UA (2.25 × 10^−9^ mol^2^L^−2^ at 37 °C), K_a1_ is the first dissociation constant of UA (4.2 × 10^−6^ mol/L at 37 °C) [28], *γ*_HU_− is the monovalent ion activity coefficient of HU^−^ at the characteristic ionic strength of urine (~0.75) [29], and $c\left(H_{2}U\right)$ is the molar concentration of UA in the sample [29]. 

### 4.6. UA Crystallization Test

**Figure 9:**
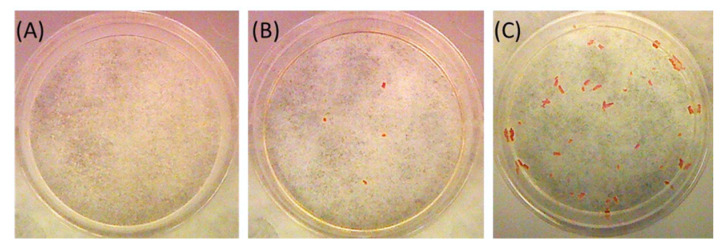
UA crystallization test results after incubation of 5 mL of different urine samples for 24 h. (**A**) No UA crystals (negative result); (**B**,**C**) UA crystals with typical orange color (positive results).

UA crystallization was determined by measuring the formation of UA crystals in polystyrene non-treated culture dishes (Corning, NY, USA), in which 5 mL of urine was maintained for 24 h at room temperature and then carefully removed via aspiration with a pipette. This test is a simplified version of the Risk for UA Crystallization (RUAC) test [30]. A result was considered positive when UA crystals formed in the dish and negative when the dish contained no crystals (Figure 9).

### 4.7. Statistics

Intergroup comparisons (Δ induction time between compounds) and intragroup comparisons (Δ induction time between different concentrations of the same compound) were performed using a one-way ANOVA test. A two-tailed *p*-value less than 0.05 was considered statistically significant. Statistical analyses were performed using SPSS version 23.0 (SPSS Inc., Chicago, IL, USA).

## 5. Conclusions

Theobromine and other methylxanthines that lacked a substituent at position 1 inhibited UA crystallization, but other methylxanthines did not have this effect. Individuals with clinical parameters that favored UA crystallization did not develop UA crystals when theobromine and its metabolites were in the urine at high levels. Thus, theobromine and its metabolites reduced the risk of UA lithiasis.

## Figures and Tables

**Table 1:** Composition of the stock solution of synthetic urine (adjusted to pH 5.0). Synthetic urine samples were obtained by diluting 80 mL of stock solution to a final volume of 200 mL.

Compound	Concentration (mM)
Na_2_SO_4_·10H_2_O	19.34
MgSO_4_·7H_2_O	5.92
NH_4_Cl	86.75
KCl	162.69
NaH_2_PO_4_·2H_2_O	15.45
Na_2_HPO_4_·12H_2_O	15.64
NaCl	223.31

## Data Availability

The data that support the findings of this study are available from the corresponding author [A.C.-B.], upon reasonable request.
